# CD20 expression is closely associated with Epstein–Barr virus infection and an inferior survival in nodular sclerosis classical Hodgkin lymphoma

**DOI:** 10.3389/fonc.2022.993768

**Published:** 2022-09-06

**Authors:** Xia Zhao, Yushuo Ma, Haiyan Bian, Zhihe Liu

**Affiliations:** ^1^ Department of Lymphoma, The Affiliated Hospital of Qingdao University, Qingdao, China; ^2^ Department of Medicine, Medical College of Qingdao University, Qingdao, China

**Keywords:** CD20, Epstein–Barr virus, nodular sclerosis, Hodgkin lymphoma, overall survival

## Abstract

**Background:**

Nodular sclerosis classical Hodgkin lymphoma (NSCHL) is a rare disease in which Epstein–Barr virus (EBV) and CD20 can be detected. The clinical significance of EBV infection, CD20 expression and their relationship are still unclear in NSCHL presently. The aim of this research was to systematically explore the clinical significance of EBV infection, expression of CD20 and their relationship in NSCHL.

**Methods:**

109 NSCHL patients diagnosed in Qingdao University’s Affiliated Hospital were chosen from January 2010 to July 2019, and the clinical and survival data of all patients were collected retrospectively.

**Results:**

Among 109 patients, 33 patients were assigned to the group of EBV-positives, following the results of the EBV-encoded RNA (EBER1). Compared with EBV-negative group patients, those in the group of EBV-positive were older (*P*=0.004) and their β2-microglobulin (β2-MG) levels were higher (*P*=0.006). The CD20 positivity rate in the group of EBV-positive was substantially higher than that in the EBV-negative group (54.5% vs 27.6%, *P*=0.007). Among 109 patients, EBV+ and CD20+ double positive patients acquired the least overall survival (OS), and patients with EBV- and CD20- double negative had the best OS (*P* < 0.001). Although old age, gender, EBV infection and CD20 positive were the risk factors for OS in NSCHL, multivariate analysis showed that CD20 positivity was the only characteristic that showed to be an independent risk factor for OS in NSCHL patients.

**Conclusion:**

CD20 was found to be strongly expressed in NSCHL patients who had been infected with EBV, and it was found to be an independent risk factor for NSCHL patients’ survival.

## Introduction

Hodgkin lymphoma (HL) is a group of highly heterogeneous lymphoid neoplasia that originates from B cells, which generally has good clinical prognosis. The World Health Organization has a classification system for lymphoid neoplasms (2016 Version) (1); there are two main pathological subtypes of HL: classical HL (CHL) and HL dominated by nodular lymphocytes. CHL is further classified into the following four types: lymphocyte-rich, lymphocyte depletion, mixed cellularity, and nodular sclerosis. In China, nodular sclerosis CHL (NSCHL) is a common pathological subtype (2, 3).

EBV is a human herpesvirus with double-stranded DNA that belongs to the gamma herpesvirus subfamily. It has been reported that 90%-95% of people will be infected with the virus in their lifetime (4). At present, the development of several malignant tumors, including lymphomas, has been linked to EBV infection (5). However, the clinical significance of EBV infection and its exact pathogenesis in CHL patients remains unclear for now (6–10).

CD20 is a hallmark of B lymphocyte cells, but less than 40% of CHL patients are CD20 positive. At present, the clinical prognostic value of CD20 in patients with NSCHL remains controversial. In this study, to systematically investigate the clinical significance of EBV infection, CD20 expression and its relationship in patients with NSCHL, we retrospectively collected and analyzed the clinical data and survival data of 109 NSCHL patients admitted to Qingdao University’s affiliated hospital in the period of January 2010 to July 2019.

## Methods

### Patients

We reviewed the medical records of all NSCHL patients admitted to Qingdao University’s affiliated hospital from January 2010 to July 2019. All patients enrolled in this study satisfied the following criteria: 1) all patients were newly diagnosed and untreated adult NSCHL (age ≥ 18 years). Physicians from Qingdao University’s Affiliated Hospital’s Hematology Department diagnosed the pathologic diagnosis of all patients again; 2) Patients were given an ABVD (adriamycin, bleomycin, vinblastine, and dacarbazine) chemotherapy regimen as a first-line treatment and/or radiotherapy, and all patients received at least four cycles of this regimen according to risk stratification; 3) Clinical characteristics, laboratory data, and follow-up data of patients were available; 4) NSCHLs from central nervous system, gray zone lymphoma and immunodeficiency related lymphomas were excluded; 5) In view of the extensive differential diagnosis and high clinical relevance of EBV positive Hodgkin’s lymphoma, this study had taken some diseases as key differential diagnosis targets, such as Hodgkin-like angioimmunoblastic T-lymphomas and secondary EBV positive B cell proliferation. Patients with controversial pathological diagnosis were also excluded. In the end, 109 NSCHL patients were included in the study. The Ethical Committee of Qingdao University’s Affiliated Hospital approved this study, which was carried out in compliance with the Helsinki Declaration. Informed consent was obtained from patients.

The time from the date of NSCHL diagnosis to the date of death from any cause or the last follow-up was defined as overall survival (OS).

### The definition of EBV-positive

*In situ* hybridization was used to detect EBV-encoded small nuclear RNA (EBER) oligonucleotide in paraffin-embedded specimens recovered from NSCHL patients, as previously described (11). The patient was considered as EBV-positive if the HRS cells expressed EBER1.

### The definition of CD20-positive

Immunohistochemistry staining workups included LCA, CD3, CD15, CD19, CD20, CD30, EMA, PAX-5, BOB.1 and OCT-2. According to a previously published article, in this study, if more than 10% of the lymphoma cells were stained, the specimen was considered positive (12).

### Statistical analysis

The data was analyzed using IBM SPSS Statistics 23.0 software (IBM Corp., Armonk, NY, USA). If appropriate, chi-square test, Student’s t test, or Fisher’s exact test were used to compare clinical information and CD20 positivity rates between EBV-positive and EBV-negative patients. The Kaplan–Meier technique (Log-rank test) was used to assess the OS, and alive cases at the last follow-up were censored. The risk factors linked with OS in patients with NSCHL were investigated using Cox proportional hazards regression analysis (multivariable analysis). Differences were defined as significant when the *P* values (two sided) ≤ 0.01.

## Results

### Base information

This study eventually enrolled a total of 109 NSCHL patients. The male to female ratio was 1.8:1.0, and the median age at diagnosis for all patients was 35 years (18-77 years). Based on the EBER1 analysis results, the EBV-positive group consisted of 33 patients, while the EBV-negative group consisted of 76 patients. We analyzed the clinical information of EBV-positive and EBV-negative patients, including gender, age, clinical stage, LDH levels, β2-MG levels, ESR levels, and B symptoms, and found that age (P=0.004) and β2-MG levels (P=0.006) were significantly different between the two groups (**Table 1**).

**Table 1:** Clinical information of nodular sclerosis classical Hodgkin lymphoma in this cohort.

	EBV-positive group (n, %)	EBV-negative group (n, %)	*P* value
Gender			0.037
Male	26 (78.8)	44 (57.9)	
Female	7 (21.2)	32 (42.1)	
Age (median, range)	47 (18-77)	34 (18-59)	0.004
Clinical stage			0.467
I, II	13 (39.4)	38 (50.0)	
III, IV	20 (60.6)	38 (50.0)	
LDH levels (U/L)			0.045
≤250	14 (42.4)	48 (63.2)	
>250	19 (57.6)	28 (36.8)	
β2-MG levels (mg/L)			0.006
≤2.7	2 (6.1)	23 (30.3)	
>2.7	31 (93.9)	53 (69.7)	
ESR levels (mm/1 h)			0.083
≤15	10 (30.3)	12 (15.8)	
>15	23 (69.7)	64 (84.2)	
B symptoms			0.625
No	17 (51.5)	43 (56.6)	
Yes	16 (48.5)	33 (43.4)	

LDH, lactate dehydrogenase; β2-MG, β2-microglobulin; ESR, erythrocyte sedimentation rate.

### CD20 expression

In the EBV-positive group, 54.5% (18/33) of patients had CD20 expression; however, only 27.6% (21/76) of patients were positive for CD20 in the EBV-negative group. Thus, the EBV-positive group had significantly more patients with CD20 expression, as compared to the patients in the EBV-negative group. Based on these observations, we can conclude that CD20 expression differed significantly between the two groups (P=0.007) with almost double the number of patients with CD20 expression in EBV-positive group, relative to EBV-negative group.

### Survival

**Figure 1:**
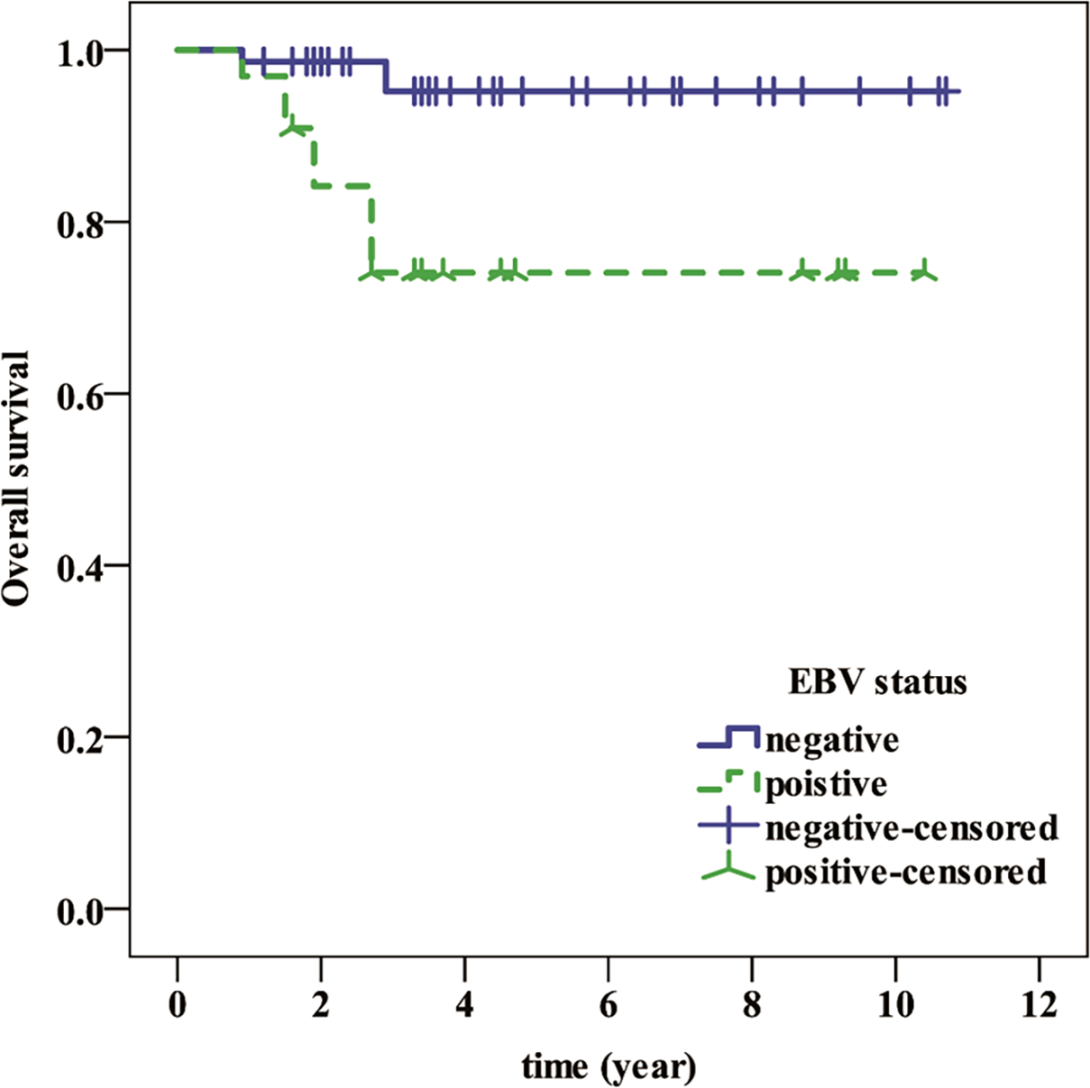
Shows the overall survival of EBV-positive and EBV-negative patients.

**Figure 2:**
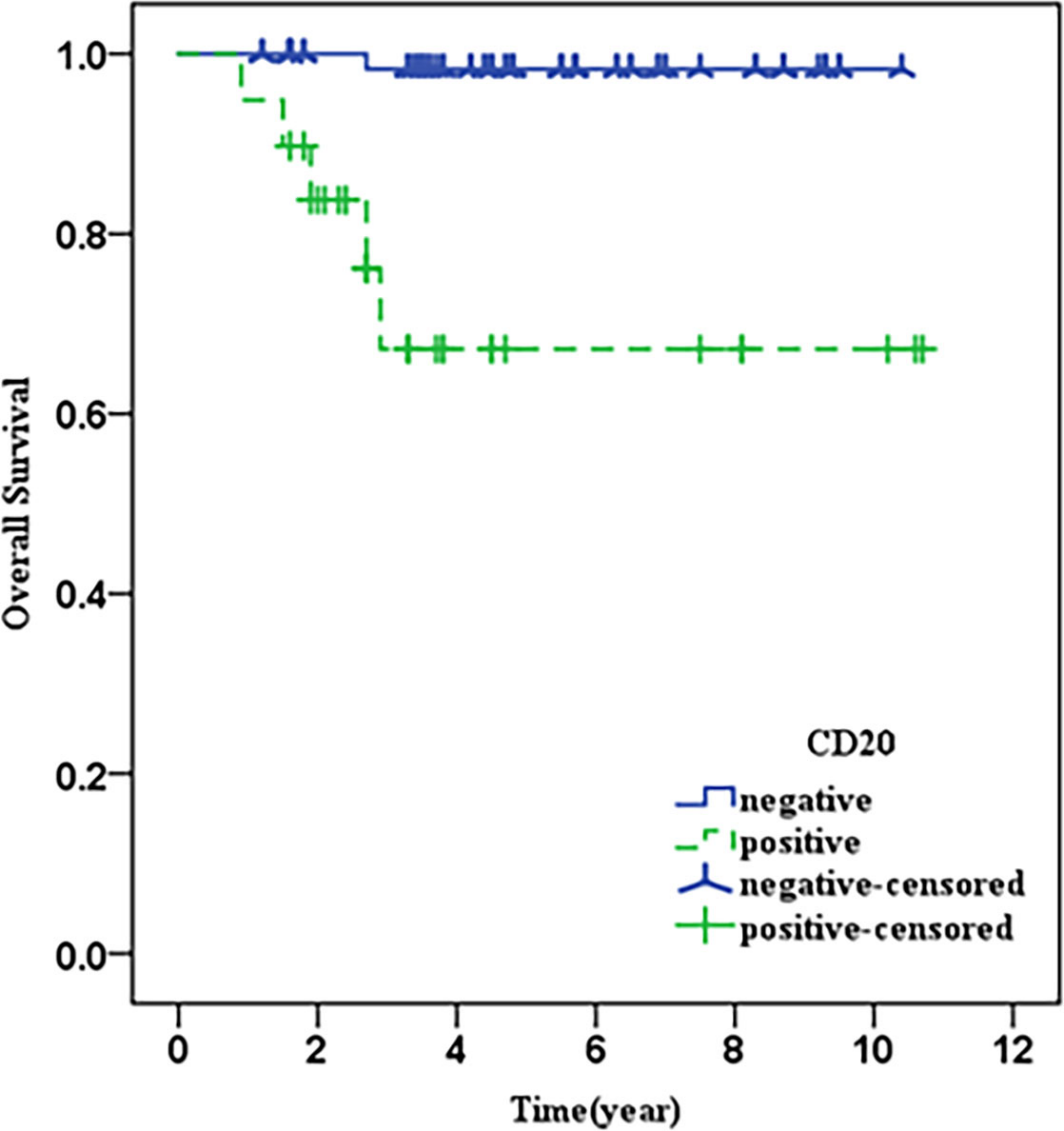
Shows the overall survival of CD20 positive and CD20 negative patients.

**Figure 3:**
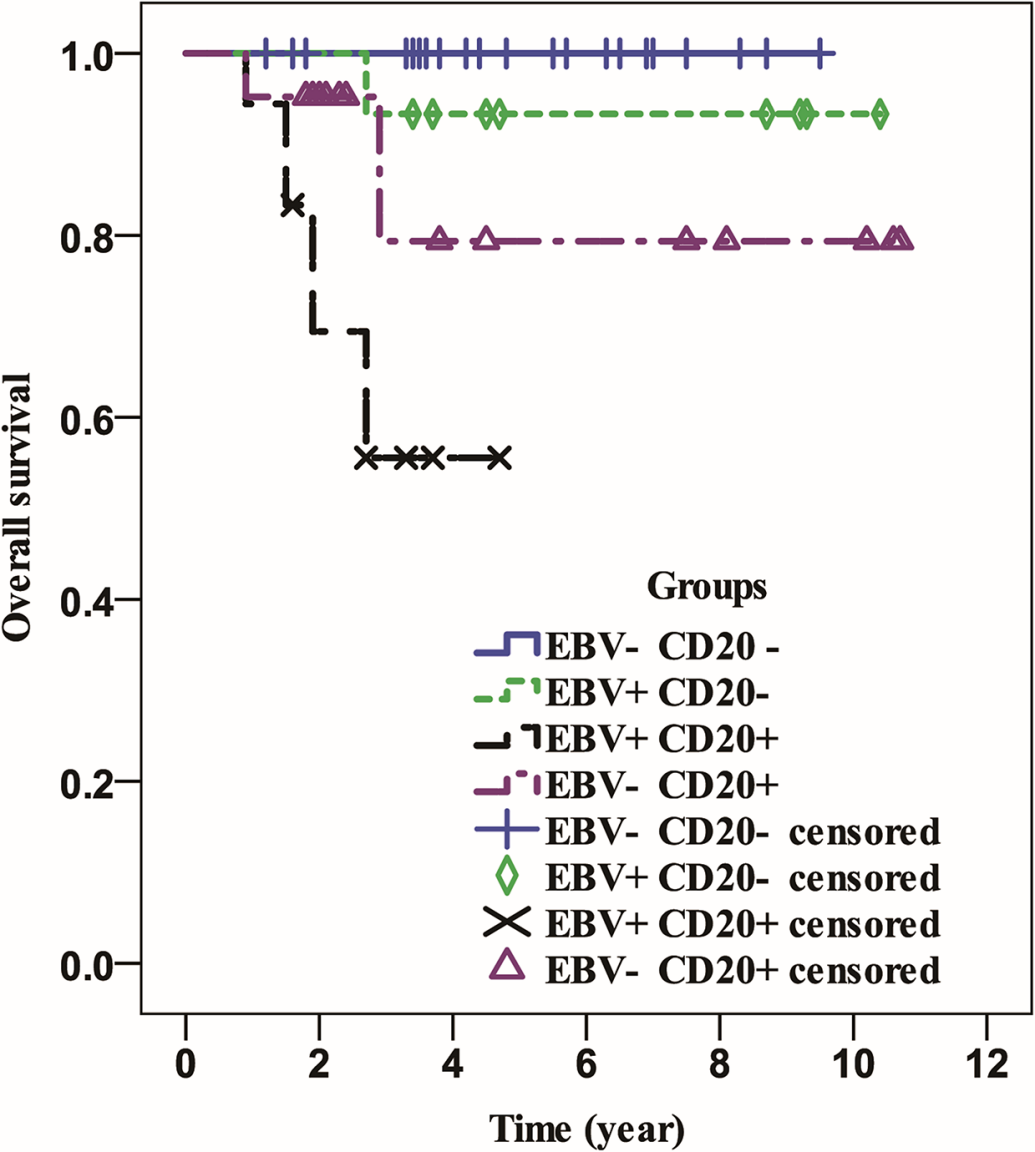
The overall survival of patients with different status of EBV infection and CD20 expression.

All patients were followed for a median of 3.8 years (range 0.9-10.7 years). Patients in the EBV-positive group had a significantly shorter OS (P=0.001) than those in the EBV-negative group (**Figure 1**), but neither group had a median OS. Similarly, whereas the median OS of CD20 positive patients was considerably lower than that of CD20 negative patients (P0.001), neither group’s median OS was attained (**Figure 2**). Then, we further explored the OS of patients with different EBV infection and CD20 expression status in our cohort. The results revealed that the patients with EBV positive and CD20 positive double positive had the worst OS, followed by patients with EBV negative combined with CD20 positive and patients with EBV positive combined with CD20 negative, and patients with double negative (EBV negative combined with CD20 negative) had the best OS (*P*<0.001) (**Figure 3**).

### Multivariate regression analysis for OS in patients with NSCHL

In this section, we firstly investigated the risk factors, including gender, age, clinical stage, LDH levels, β2-MG levels, ESR levels, B symptom, EBV status and CD20 expression. The Kaplan–Meier technique (Log-rank test) was used to calculate the odds of survival in 109 NSCHL patients, and the results revealed that age (P=0.001), EBV status (P=0.001), and CD20 expression (P0.001) were all risk factors. Then, we conducted multivariate regression analysis on the above three risk factors (age, EBV status and CD20 expression). CD20 positivity was found to be an independent risk factor for OS in NSCHL patients (**Table 2**).

**Table 2:** Multivariate regression analysis for overall survival in NSCHL patients.

	Multivariate analysis		
	HR	HR (95% CI)	*P* value
Age			
>45 years	3.205	0.840 – 12.195	0.088
EBV infection			
Positive	1.264	0.297-5.376	0.752
CD20 expression			
Negative	17.544	2.088-142.857	0.008

CI, confidence interval; HR, hazard ratio.

## Discussion

CHL is a hematological malignant cancer that accounts for less than 10% of lymphomas in Asia, including China (13, 14). Hodgkin and Reed-Sternberg cells, a hallmark of CHL, disperse in the reactive inflammatory microenvironment of lymphoid tissues. Although it has been reported that up to 40% of HL patients had EBV infection, the function of EBV infection in the clinical outcome of HL patients is still debated (15). Some studies revealed that EBV infection could be a good prognostic factor for HL patients (16, 17). However, it was also reported that EBV infection could be an adverse risk factor for worse survival times in HL patients in some articles (18). In addition, other studies have displayed that there were no significant effects on the clinical prognosis of HL patients infected with EBV (9, 19). In this study, in NSCHL patients, EBV infection was a poor prognostic factor for survival. We considered that this conclusion may be associated with following two factors. First, some studies have confirmed that CHL patients with age more than 50 years had a poorer clinical prognosis, and the median age of patients in EBV-positive group was 13 years older than that in EBV-negative group (47 years versus 34 years) (20, 21). Secondly, CD20 positivity was significantly greater in the EBV-positive group than in the EBV-negative group, and CD20 positivity was confirmed as an independent risk factor for OS in NSCHL patients in this study.

CHL is a malignant tumor derived from B lymphocytes. To the best of our knowledge, the vast majority of CHL cells express CD15 and CD30 antigens. However, only 20% ~ 40% of CHL patients have CD20 positive cells (22). To date, the role of CD20 positive in the clinical prognosis of CHL is still unclear. Greaves et al. discovered that there was no statistical significance between CD20 positive and the prognosis of patients with HL (23). However, some studies demonstrated that CD20 positive was closely associated with favorable OS and PFS in CHL patients (24, 25). Other studies showed that the clinical prognosis of CHL patients with CD20 positive was poorer (26, 27). In our study, CD20 positive was a poor prognostic factor for the OS NSCHL patients. We speculate that age may be an important factor associated with the poor prognosis of NSCHL CD20-positive patients, as the median age was 12 years older for NSCHL CD20-compared to CD20-negative NSCHL patients.

At present, chemotherapy and radiotherapy are still the first-line therapy strategies for CHL patients. The vast majority of CHL patients achieve complete remission after the initial treatment. However, 34% of CHL patients with stage III/IV disease and 15% of CHL patients with stage I/II disease eventually experience relapse after the first-line treatment regimens (28). For those patients, with the development of medical technology, novel treatment strategies can be chosen at the time of definite diagnosis, such as checkpoint blocking therapy, chemoimmunotherapy and anti-EBV proteome antibody therapy (29–32). CD20 positivity was shown to be significantly greater in EBV-positive patients with NSCHL in this study. Therefore, we propose that clinical efficacy to therapy in EBV-positive patients with NSCHL may be further improved by incorporating an anti-CD20 monoclonal antibody (rituximab) into the first-line chemotherapy regimen.

The study has some limitations that need to be addressed. To begin with, this is a retrospective, single-centered study. Secondly, the sample size of this study is small, particularly for EBV-positive patients with NSCHL. Therefore, in order to verify the reliability of the conclusions of this study, we will further increase the sample size of EBV-positive patients with NSCHL by conducting a multi-center study in China in the future.

In conclusion, EBV-positive NSCHL may be a distinct disease entity characterized by advanced age, higher levels of β2-MG, and dismal prognosis. CD20 is highly expressed in EBV-positive patients with NSCHL, In NSCHL patients, this was an independent risk factor for OS.

## Data availability statement

The original contributions presented in the study are included in the article/Supplementary Material. Further inquiries can be directed to the corresponding author.

## Ethics statement

The studies involving human participants were reviewed and approved by The Ethical Committee of Qingdao University’s Affiliated Hospital. The patients/participants provided their written informed consent to participate in this study.

## Author contributions

XZ and YM collected data and performed analysis. HB analyzed data. ZL supervised study and wrote first draft. XZ, YM, HB and ZL edited manuscript. All authors contributed to the article and approved the submitted version.

## Funding

This study was funded by the China Postdoctoral Science Foundation (Grant No.2020M682128 to XZ) and the project of the Affiliated Hospital of Qingdao University (Grant No. QDFY201929 to ZL).

## Acknowledgments

We would like to show our deepest gratitude to the professors from division of Hematopathology in the Affiliated Hospital of Qingdao University, who had provided us with valuable guidance in pathologic diagnosis.

## Conflict of interest

The authors declare that the research was conducted in the absence of any commercial or financial relationships that could be construed as a potential conflict of interest.

## Publisher’s note

All claims expressed in this article are solely those of the authors and do not necessarily represent those of their affiliated organizations, or those of the publisher, the editors and the reviewers. Any product that may be evaluated in this article, or claim that may be made by its manufacturer, is not guaranteed or endorsed by the publisher.

## Abbreviations

b2–MG, b2–microglobulin; CHL, classical HL; CI, confidence interval; EBV, Epstein–Barr virus; ESR, erythrocyte sedimentation rate; HL, Hodgkin lymphomas; HR, hazard ratio; LDH, lactate dehydrogenase; NSCHL, Nodular sclerosis classical Hodgkin lymphoma; OS, Overall survival.
